# Computed tomographic diagnosis of aortocaval fistula

**DOI:** 10.4102/sajr.v22i1.1363

**Published:** 2018-09-12

**Authors:** Navdeep Singh, Aneesh Mangalasseril Kuriakose, Regi AN George, Shirish Vaidya

**Affiliations:** 1 Department of Radiodiagnosis, Jubilee Mission Medical College and Research Institute, India

## Abstract

Aortocaval fistula is an abnormal vascular communication between an aortic aneurysm and the inferior vena cava. The condition is very rare and life threatening with high mortality. This article reports a case of aortocaval fistula in a 76-year-old male diagnosed using computed tomography. Computed tomography, being a non-invasive investigation, ensures early diagnosis and can help in prompt surgical or interventional radiological management which can be life-saving.

## Introduction

Aortocaval fistula (ACF) is a rare complication of abdominal aortic aneurysm (AAA) and is associated with a high mortality rate. It is seen in about 4% of surgeries for ruptured aneurysms.^1^ Early preoperative diagnosis can aid in dedicated preparation for surgical or interventional radiological treatment. While evaluating an AAA on computed tomography (CT), certain imaging findings can help in diagnosing ACF.

## Case report

A 76-year-old male patient presented with severe abdominal pain associated with breathing difficulty. On examination, the patient was hypotensive with tachycardia and a pulsatile abdominal mass. Emergency ultrasonography (US) revealed an infrarenal aortic aneurysm with peripheral thrombus.

For further evaluation of the extent of aneurysm, emergency CT angiography was performed using a dual slice helical CT scan (General Electric, Milwaukee). A total of 100 mL of non-ionic contrast medium iopamidol (Lek-pamidol, India), was injected in the right antecubital vein at a flow rate of 4 mL/s. Image acquisition was started after a delay of 22 s. The CT parameters were 120 kV, 120 mA, 2 mm slice thickness and 1.5 pitch. The area of coverage extended from the sternal angle to the inguinal region.

**FIGURE 1:**
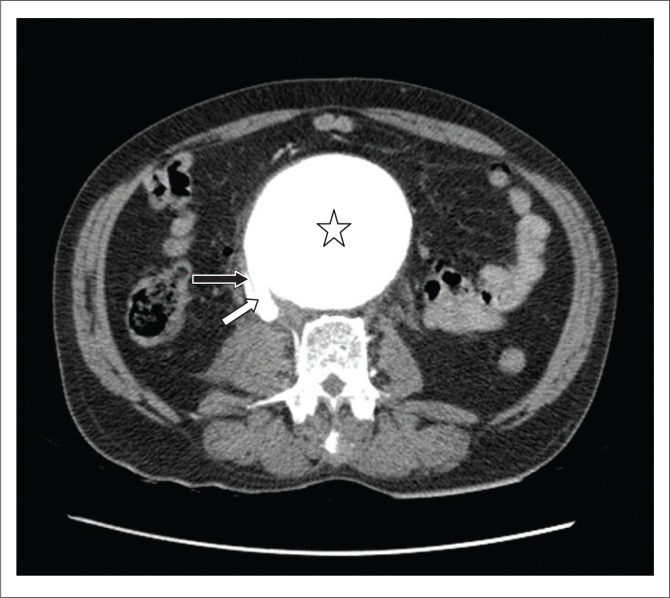
Axial contrast-enhanced computed tomography showing a large infrarenal aneurysm (star) having a fistulous communication (white diagonal arrow) on its right posterolateral wall with inferior vena cava (black arrow).

The scan showed a large infrarenal fusiform AAA with a partially thrombosed periphery. Active extra luminal extravasation of contrast was seen from the right lateral wall, into the peritoneal cavity (Figures 1 and 3a).

**FIGURE 2:**
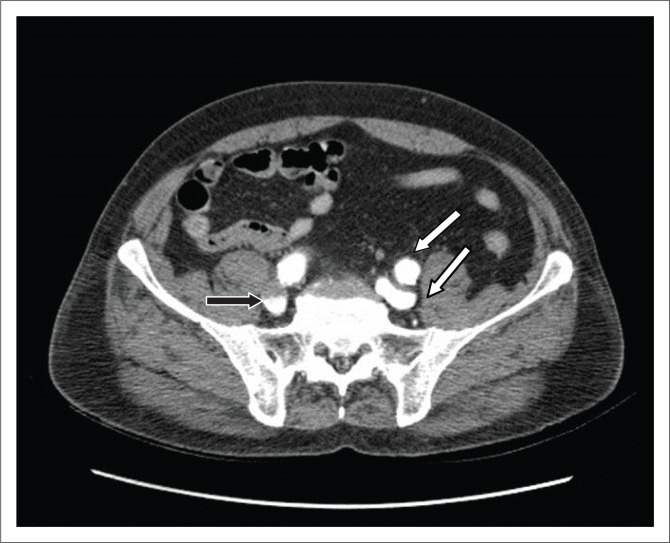
Axial contrast-enhanced computed tomography scan during the arterial phase showing opacification of the common iliac arteries and veins bilaterally, with similar attenuation (white arrows); layering of contrast was observed in the iliac veins (black arrow).

**FIGURE 3:**
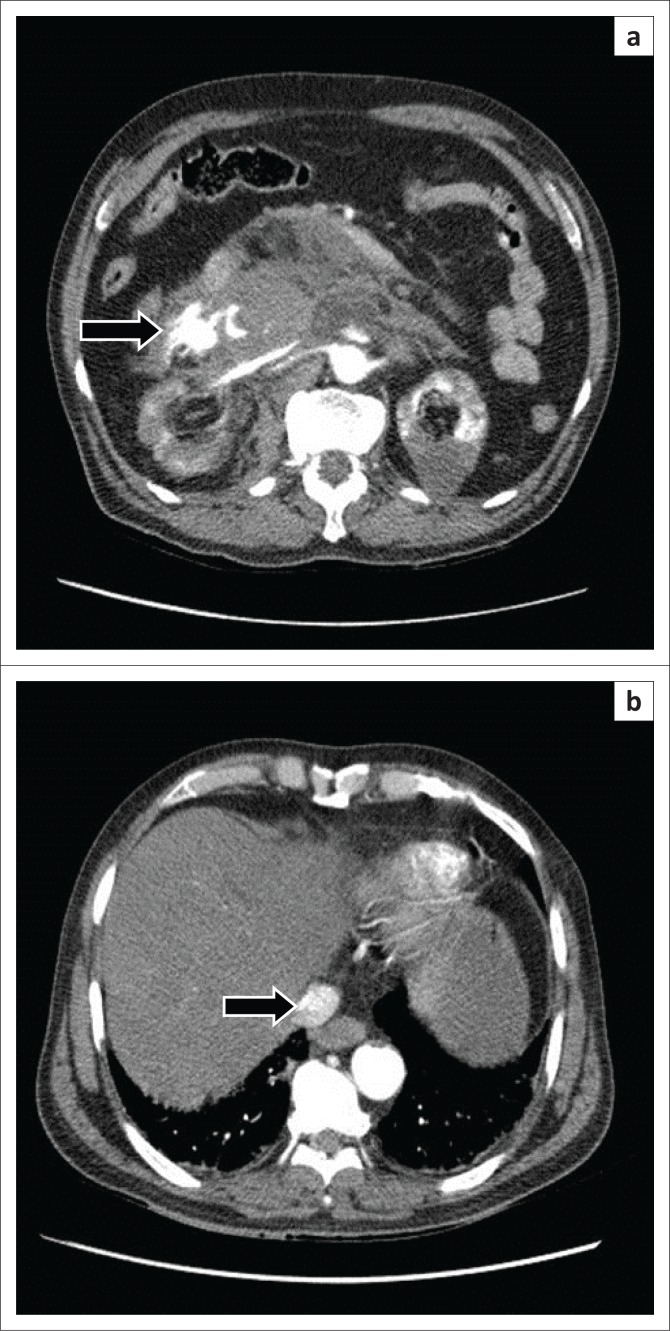
(a) Axial contrast-enhanced computed tomography scan demonstrating active extravasation of contrast into the peritoneal cavity (arrow). Contrast is also seen in the renal veins; (b) Contrast seen in the hepatic inferior vena cava (arrow) but with less attenuation (HU – 150) owing to reflux from right atrium.

There was a fistulous communication on the right posterolateral wall of aorta with the inferior vena cava (IVC), with a flap displaced into the IVC about 4.5 cm distal to the origin of the renal arteries (Figure 1). The lower abdominal IVC and iliac veins were opacified on the arterial phase with contrast medium density similar to that of the aorta (HU 350) (Figure 2). The upper abdominal IVC was compressed by the aneurysm and the hepatic portion demonstrated minimal opacification with an attenuation of 150 HU. This helped in excluding reflux of contrast from the right atrium as the cause for opacification in the lower abdominal IVC (Figure 3b).

In view of these findings, a diagnosis of a ruptured AAA with an ACF was made. The patient was planned for emergency surgery. However, he suffered a cardiac arrest before being shifted to the operating room and could not be revived.

## Discussion

Abdominal aortic aneurysms mostly rupture into the retro-peritoneum or peritoneal cavity. Aortocaval fistula is a rare complication of an aortic aneurysm which can occur spontaneously in 1% of patients or along with rupture in 4% of patients.^1^ Men in the sixth to seventh decade are commonly affected. According to a recently proposed mechanism, development of ACF involves pressure necrosis of the aortic wall from the aortic aneurysm. This leads to severe periaortic inflammation causing adherence of an aneurysm to the IVC, subsequently eroding into it. Various risk factors include atherosclerosis, mycotic aneurysm, syphilis, polyarteritis nodosa and connective tissue disorders like Marfan syndrome and Ehlers–Danlos syndrome.^2^

The clinical presentation depends upon the acuteness of the condition and the presence of other associated findings, like rupture and dissection. A palpable pulsatile mass with a continuous bruit in a haemodynamically stable patient suggests an unruptured AAA, whereas a ruptured aneurysm presents with severe abdominal pain, hypotension and shock.^3^ Other symptoms include shortness of breath, cold extremities, cyanosis, haematuria and pulmonary oedema. Long-standing fistulas often present with features of high output cardiac failure.^1,4^

Preoperative diagnosis is important in planning the operative management and preventing further complications. According to one study, mortality rate in open surgical repair of ACF was around 30%, mainly from excessive blood loss, but it was significantly lower if a preoperative diagnosis was available.^5^

Ultrasound examination can be the initial imaging modality to diagnose AAA but has a very limited role in diagnosing ACF. Multidetector CT angiography is the preferred investigation. Simultaneous presence of contrast in the aorta and IVC during the arterial phase is the most important diagnostic sign and this was demonstrated in our patient. Quite often, the aneurysm compresses the IVC, thereby making it difficult to evaluate this sign. The screening of the iliac veins and renal veins is very significant in such situations as they may be opacified synchronously and with similar attenuation to the aorta. The reflux of contrast from the right atrium should not be mistaken as ACF, as it is of less attenuation. The presence of a fistulous communication between the aorta and IVC is not seen in all cases but was demonstrated in our patient. Additional signs which help in diagnosing an ACF are the loss of the fat plane between the aorta and IVC, poor perfusion of kidneys and presence of aneurysmal rupture.^6^ Aortography is confirmative in diagnosing the ACF.

**FIGURE 4:**
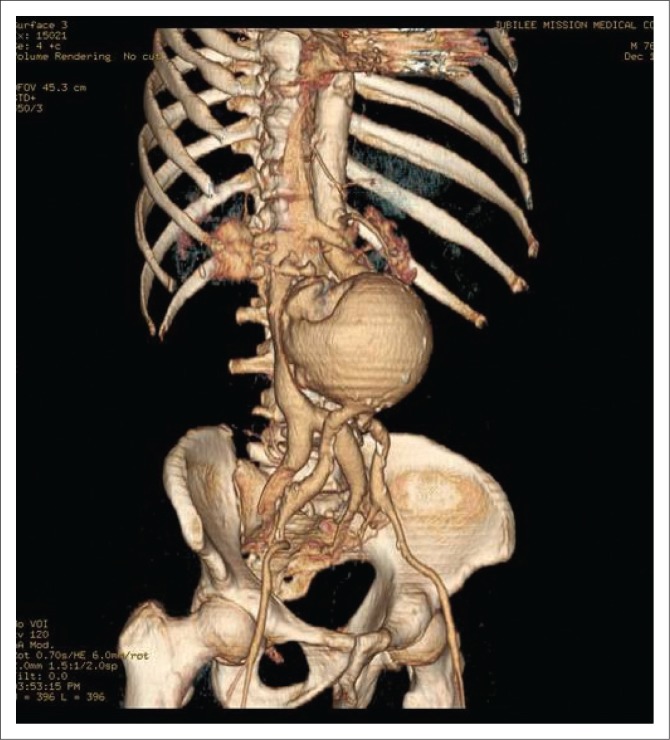
Volume-rendered arterial phase image displays the opacified aneurysm, iliac veins and inferior vena cava.

Successful treatment of ACFs depends upon early preoperative diagnosis, proper operative planning, haemodynamic stabilisation and prevention of pulmonary embolism. Open surgical repair or endovascular repair forms the mainstay of treatment. Endovascular repair has the advantage of less blood loss as compared to the open repair with equal success.^5^

## Conclusion

In conclusion, ACF is a rare complication of AAA, associated with a high mortality rate. Timely diagnosis is possible by pursuing certain findings at CT imaging which can aid in proper surgical or endovascular planning.
